# Application of sulfur SAD to small crystals with a large asymmetric unit and anomalous substructure

**DOI:** 10.1107/S2059798322005848

**Published:** 2022-07-14

**Authors:** Tung-Chung Mou, Baisen Zeng, Tzanko I. Doukov, Stephen R. Sprang

**Affiliations:** a Center for Biomolecular Structure and Dynamics, University of Montana, Missoula, MT 59812, USA; b Division of Biological Sciences, University of Montana, Missoula, MT 59812, USA; c Macromolecular Crystallography Group, Stanford Synchrotron Radiation Light Source, SLAC National Accelerator Laboratory, Stanford University, Stanford, CA 94309, USA

**Keywords:** sulfur SAD phasing, data scaling, single-wavelength anomalous dispersion, sulfur substructure determination, large asymmetric unit, Ric-8A

## Abstract

The application of sulfur single-wavelength anomalous dispersion (S-SAD) to determine the crystal structures of macromolecules can be challenging if the asymmetric unit is large, the crystals are small, the size of the anomalously scattering sulfur structure is large and the resolution at which the anomalous signals can be accurately measured is modest. Here, as a study of such a case, approaches to the SAD phasing of orthorhombic Ric-8A crystals are described. The structure of Ric-8A was published with only a brief description of the phasing process [Zeng *et al.* (2019 ▸), *Structure*, **27**, 1137–1141]. Here, alternative approaches to determining the 40-atom sulfur substructure of the 103 kDa Ric-8A dimer that composes the asymmetric unit are explored. At the data-collection wavelength of 1.77 Å measured at the Frontier micro-focusing Macromolecular Crystallography (FMX) beamline at National Synchrotron Light Source II, the sulfur anomalous signal strength, |Δ_ano_|/σΔ_ano_ (*d*′′/sig), approaches 1.4 at 3.4 Å resolution. The highly redundant, 11 000 000-reflection data set measured from 18 crystals was segmented into isomorphous clusters using *BLEND* in the *CCP*4 program suite. Data sets within clusters or sets of clusters were scaled and merged using *AIMLESS* from *CCP*4 or, alternatively, the *phenix.scale_and_merge* tool from the *Phenix* suite. The latter proved to be the more effective in extracting anomalous signals. The *HySS* tool in *Phenix*, *SHELXC*/*D* and *PRASA* as implemented in the *CRANK*2 program suite were each employed to determine the sulfur substructure. All of these approaches were effective, although *HySS*, as a component of the *phenix.autosol* tool, required data from all crystals to find the positions of the sulfur atoms. Critical contributors in this case study to successful phase determination by SAD included (i) the high-flux FMX beamline, featuring helical-mode data collection and a helium-filled beam path, (ii) as recognized by many authors, a very highly redundant, multiple-crystal data set and (iii) the inclusion within that data set of data from crystals that were scanned over large ω ranges, yielding highly isomorphous and highly redundant intensity measurements.

## Abstract 2

Multiple approaches to data merging and scaling and anomalous substructure determination were used to solve the structure of a large asymmetric unit from weakly diffracting crystals by sulfur SAD.

## 1. Introduction

Despite recent advances in synchrotron hardware, data-collection strategies and crystallographic software packages, the *de novo* phasing of macromolecular crystal structures by sulfur single-wavelength anomalous dispersion (S-SAD) from native sulfur atoms can be challenging (Liu & Hendrickson, 2015 ▸, 2017 ▸; Rose *et al.*, 2015 ▸; Terwilliger *et al.*, 2016 ▸; Olieric *et al.*, 2016 ▸; Akey *et al.*, 2016 ▸; Weiss, 2017 ▸). Here, we describe strategies to determine the anomalously scattering sulfur substructure of Ric-8A, which was a necessary step towards the solution of its structure by SAD. In a previous publication we described the structure of Ric-8A, but only briefly the methods by which it was determined (Zeng *et al.*, 2019 ▸).

The weak anomalous scattering signal (Bijvoet ratio = ∼1% of the total reflection intensities) from sulfur atoms in proteins limits its utility for phase determination. The anomalous signal is a function of the square root of the ratio of the number of unique reflections, and hence the resolution, to the number of atoms in the anomalous substructure (Terwilliger *et al.*, 2016 ▸). Successful application of S-SAD may require (i) the accurate collection and merging of highly redundant and isomorphous data sets with quantitatively strong signal-to-noise ratios, often using strategies tailored to individual synchrotron sites, and (ii) the finding of sulfur substructures using optimized parameters in crystallographic software packages to maximize sulfur anomalous signals (Liu & Hendrickson, 2015 ▸, 2017 ▸; Olieric *et al.*, 2016 ▸; Akey *et al.*, 2016 ▸; Bunkóczi *et al.*, 2015 ▸).

Several recently published reviews have addressed major advances in synchrotron hardware and crystallographic software to reduce systematic errors that obscure anomalous differences arising from sulfur substructures (Liu & Hendrickson, 2015 ▸, 2017 ▸; Terwilliger *et al.*, 2016 ▸; Olieric *et al.*, 2016 ▸; Akey *et al.*, 2016 ▸; Rose *et al.*, 2015 ▸; Hendrickson, 2014 ▸). As recent generations of synchrotron beamlines have been constructed with novel hardware configurations, it may be important to design a data-collection strategy that is beamline-specific to acquire highly precise and redundant S-SAD anomalous data sets. From crystal-harvesting loop selection to data processing, every step is crucial for collecting useful S-SAD anomalous data. However, many practices are generally applicable to all beamlines.

Currently, a common approach is to measure S-SAD anomalous data at 6000–7000 eV, at which the sulfur anomalous signal (*f*′′ of 0.8 e^−^) is significant but absorption is low, with a fast and large-area photon-counting detector. Fast-readout photon-counting detection is especially useful when data can be collected in a shutterless and fine-sliced oscillation mode. Recently, it has been possible to measure S-SAD data at longer wavelengths near the sulfur *K* edge at very special­ized synchrotron beamlines. One example is the I23 beamline at Diamond Light Source, UK, where evacuation of the space between the sample and detector reduces air absorption at low energy and the use of a unique semi-cylindrical, large-area, pixel-array detector affords access to diffraction at high 2θ angles (Weiss, 2017 ▸; Wagner *et al.*, 2016 ▸).

It is generally agreed that sulfur anomalous signals can be boosted by collecting high-multiplicity data sets within an optimal resolution range. However, it may not be possible to obtain such data sets from a very small crystal that is subject to radiation damage or belongs to a low-symmetry space group (Klinke *et al.*, 2015 ▸). In such cases, it may be possible to merge data sets collected from multiple isomorphous crystals. On the other hand, several approaches to mitigate radiation damage have been adopted in synchrotron data-collection strategies. For example, the helical data-collection mode implemented at several micro-focus beamlines allows the collection of oscillation images while translating a crystal along a defined collection path parallel to its long axis to reduce radiation damage (Polsinelli *et al.*, 2017 ▸)

If data sets from multiple crystals are required to obtain suitably accurate intensity measurements, merging and scaling thousands of frames with millions of reflections from multiple, marginally isomorphous crystals can present a challenge for S-SAD phasing. A cluster-based analysis has been used to prioritize individual unmerged data sets based on their divergence in unit-cell parameters and reflection quality, which includes the errors in measurement. This methodology has been incorporated into data-scaling and averaging programs, such as *BLEND* in *CCP*4 (Foadi *et al.*, 2013 ▸) and *phenix.scale_and_merge* (Terwilliger *et al.*, 2016 ▸). After obtaining a merged data set that optimizes anomalous signals at the highest resolution, locating the atoms of the sulfur substructure appears to be the most challenging task in solving the S-SAD phasing problem. The dual-space direct method implemented in *SHELXD* (Sheldrick, 2010 ▸) refines substructure positions and phases by recurrently alternating reciprocal-space phase refinement and density modification. The *phenix.hyss* tool (Grosse-Kunstleve & Adams, 2003 ▸; Bunkóczi *et al.*, 2015 ▸) uses both dual-space substructure completion and a correlation-based scoring procedure to find the anomalously scattering substructure, as well as SAD likelihood function-based gradient maps to complete partial substructures from the anomalous difference Patterson function and the same function to evaluate potential solutions. More recently, *PRASA* was introduced to implement a relaxed averaged alternating reflections phase-retrieval algorithm to extract the positions of anomalous scatterers from the anomalous difference data (Skubák, 2018 ▸).

Many of the hundreds of structures that have been solved by S-SAD were determined from crystals with relatively small (<25 kDa) asymmetric units that diffract to *d*-spacings beyond 2 Å, factors that are associated with strong diffraction and small sulfur substructures (Rose *et al.*, 2015 ▸; Gorgel *et al.*, 2015 ▸). For moderately or weakly diffracting crystals with large asymmetric units and sulfur substructures, which may also be subject to significant radiation decay, it becomes essential to combine all possible optimization methods to acquire accurate and highly redundant data sets that afford quantitation of anomalous differences. Moderate-to-weakly diffracting crystals require much longer exposure times to obtain anomalous data with sufficient redundancy to afford acceptable signal-to-noise ratios. One successful example was reported by Smith and coworkers, in which data sets from 28 crystals were merged to obtain the accurate sulfur anomalous signal at 4 Å resolution required to determine the flavivirus NS1 structure to 3 Å resolution (Akey *et al.*, 2016 ▸); another described the extraction of a sulfur substructure using data to 7 Å resolution from 32 crystals with phase extension to 3.5 Å resolution (El Omari *et al.*, 2014 ▸). Data collection from several crystals each in multiple orientations has also proven useful (Olieric *et al.*, 2016 ▸). Here, we summarize our experience with S-SAD phasing in such a case: to determine the structure of an asymmetric unit containing two 51 kDa domains of the protein Ric-8A using the brilliant, micro-focused beam at the tunable Frontier micro-focusing Macromolecular Crystallography (FMX) beamline at National Synchrotron Light Source II (NSLS II; Schneider *et al.*, 2021 ▸)

Ric-8A plays essential roles in cells as both a chaperone and a guanine nucleotide-exchange factor for α subunits of heterotrimeric G proteins (Tall *et al.*, 2003 ▸; Thomas *et al.*, 2011 ▸; Chan *et al.*, 2013 ▸). Since there are no homologs of Ric-8A with known structure, molecular-replacement phasing was not an option at the time that the structure was determined. Further, selenomethionine-derivatized Ric-8A only forms microcrystals, and native Ric-8A crystals are highly sensitive to heavy metals. Thus, we sought to obtain phase information from the anomalous signals arising from the sulfur atoms in the nine cysteine and ten methionine residues in each molecule of Ric-8A, which account for 4.2% of the 904 residues in the asymmetric unit. None of the cysteine residues is involved in a disulfide bond. While the best native crystals diffract to 2.5 Å resolution using X-rays of wavelength 1.77 Å, we show that sulfur anomalous data can only be accurately measured to 3–3.4 Å resolution. A survey of the PDB reveals that Ric-8A represents one of about ten crystal structures comprising more than 100 kDa per asymmetric unit (two molecules per asymmetric unit, total 102.2 kDa) that have been determined by native anomalous scattering from light atoms (*Z* < 15). We discuss data-collection strategies using the high-precision crystal-positioning hardware and control software at the FMX beamline, and the merging and scaling procedures that led to successful structure determination of Ric-8A by S-SAD.

## 2. Materials and methods

### 2.1. Crystallization of Ric-8A

**Figure 1:**
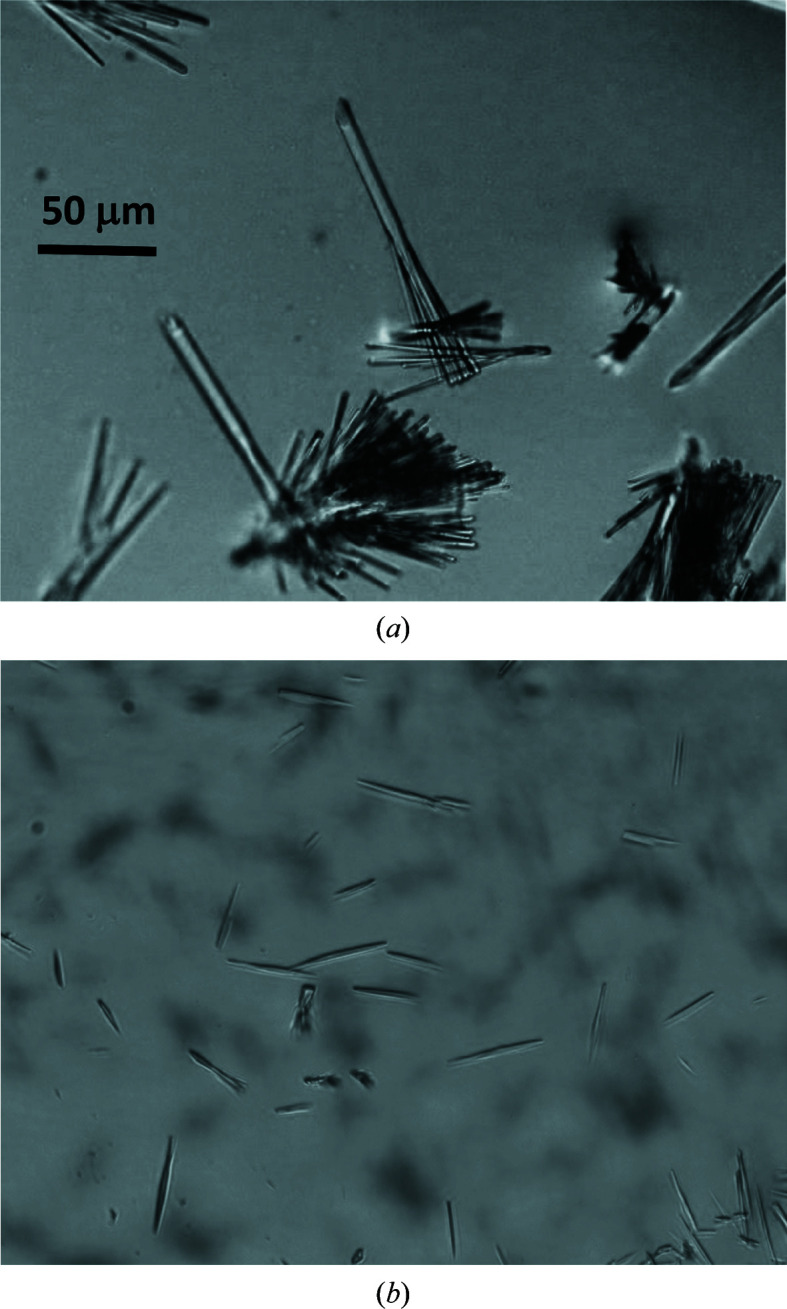
Representative (*a*) phosphorylated and (*b*) unphosphorylated Ric-8A (1–452) crystals. The scale of the two panels is the same.

The expression and purification of the N-terminally phosphorylated 452-residue Gα binding domain of rat Ric-8A has been described elsewhere (Zeng *et al.*, 2019 ▸). Briefly, initial crystallization experiments were performed using a Gryphon robot (Art Robbins, California, USA) to screen over 1000 conditions using commercially available kits by mixing equal amounts of reservoir solution with phosphorylated or unphosphorylated Ric-8A protein at concentrations as high as 75 mg ml^−1^. Small needle-like crystals were observed in conditions from The PEGs II Suite (Qiagen) at 20°C after 72 h. The reservoir solutions from the initial hits consisted of 0.2 *M* lithium sulfate, 0.1 *M* Tris buffer pH 8.0 and 25–30% PEG 4000 or PEG 5000 MME. Crystal quality was improved by using a 3:1 ratio of phosphorylated Ric-8A protein and reservoir solutions consisting of 0.2 *M* lithium sulfate, 0.1 *M* Tris or HEPES buffers pH 7–9 and 20–30% PEG 3350. The larger Ric-8A crystals, measuring 50–250 µm in the longest dimension and 5–20 µm in cross section (Fig. 1 ▸), were observed after 2–3 weeks of incubation time. Prior to mounting, crystals were harvested in a cryoprotection solution containing 20–25%(*v*/*v*) PEG 400 or oil-based cryoprotectant (Paratone-N) and then rapidly plunged into liquid nitrogen.

### 2.2. Crystal mounting

To minimize systematic errors from sample vibration during data collection, we used a 20 µm nylon crystal-mounting CryoLoop (Hampton Research) to harvest Ric-8A crystals. The thick nylon loops decrease the mechanical vibration from exposure to the N_2_ cryostream, which improves data quality, especially when data are derived from merging multiple data sets. The sample loop was mounted on a goniometer with cryocooling capability to minimize radiation damage (Garman & Owen, 2006 ▸; Teng & Moffat, 2000 ▸).

### 2.3. Data collection

18 data sets were recorded at 100 K and a wavelength of 1.7712 Å (7000 eV) using the helical data-collection method (Polsinelli *et al.*, 2017 ▸) on the micro-focusing FMX beamline at NSLS II equipped with an EIGER 16M pixel-array detector with a 133 Hz framing rate. The crystal-to-detector distance was set to 200, 175 or 150 mm according to the highest *d*-spacings at which diffraction was observed, affording the collection of data at resolutions ranging from 2.67 to 2.23 Å at the detector edge. Crystals were irradiated with a 10 × 10 µm beam at 10% attenuation of a flux of ∼5.0 × 10^12^ photons s^−1^ in a helium flight path. Data were collected with a thin-slice oscillation range (0.1–0.2° per image) at 0.1–0.2 s exposure per image for a total rotation of 360–5760° about the φ axis per data set (Table 1 ▸). The a* axis was inclined 3–15° to the φ axis for ten of the 18 data sets and within a 20–50° angle to φ for the remaining eight. Over all 18 crystals, more than 23 500° of data were measured. The data sets were processed by *XDS* (Kabsch, 2010 ▸) in space group *P*1. Analysis of the data using *POINTLESS* (Evans & Murshudov, 2013 ▸) in the *CCP*4 software package (Winn *et al.*, 2011 ▸) confirmed the space-group assignment as *P*2_1_2_1_2_1_. Each of the 18 unmerged data sets produced by *XDS* has been deposited in the PDB in *CCP*4 mtz format, associated with PDB entry 6mng, with the filename *X*-XDS.mtz, where *X* is the data-set name shown in column 1 of Table 1 ▸.

### 2.4. Data reduction, phase determination and model building

Parameters describing the 18 data sets obtained from 14 crystals (Table 1 ▸) were computed using *AIMLESS* (Evans & Murshudov, 2013 ▸) in the *CCP*4 software package, the *phenix.anomalous_signal* tool and *SHELXC* (Sheldrick, 2010 ▸). The *BLEND* suite (Foadi *et al.*, 2013 ▸) was used to cluster data sets and scale them using *AIMLESS*. The *phenix.scale_and_merge* and *phenix_scale_anomalous_signal* tools (Terwilliger *et al.*, 2016 ▸) in the *Phenix* program suite (Liebschner *et al.*, 2019 ▸) were also used to scale data sets clustered using *BLEND* and the cluster comprised of data from all crystals. In *phenix.scale_and_merge*, the data-selection parameter minimum_datafile_fraction was set to accept any data set containing at least 5% of the number of observations in the largest data set (the default value is 30%). *SHELXC*/*D*/*E* (Sheldrick, 2010 ▸), executed though the *HKL*2*MAP* graphical interface (Pape & Schneider, 2004 ▸) was used to determine the sulfur substructure from the merged data sets. The *Phenix* submodule *HySS* (Bunkóczi *et al.*, 2015 ▸) was also used to find atoms in the sulfur substructure. The *phenix.emma* program was used to correlate the candidate sulfur substructures generated using *HySS* and *SHELXD* with sulfur positions in the refined model of Ric-8A. The anomalously scattering sulfur substructure and crystallo­graphic phases were refined using the *phenix_autosol* procedure (Terwilliger *et al.*, 2009 ▸) with optimization of the positions of sulfur atoms. The twofold noncrystallographic symmetry (NCS) operator was calculated from the sulfur sites during phase refinement. Phases were extended to 2.2 Å resolution with a native data set that was measured using X-rays at a wavelength of 0.979 Å as described by Zeng *et al.* (2019 ▸) and was used to construct a partial model using the *AutoBuild* wizard (Terwilliger *et al.*, 2008 ▸). Fragments of additional main chains were constructed after iterative manual model rebuilding and refinement with the *phenix.refine* tool (Afonine *et al.*, 2012 ▸). The final refinement statistics are recorded with the description of the crystal structure (Zeng *et al.*, 2019 ▸).

## 3. Results and discussion

### 3.1. Crystallization and crystal harvesting

The largest crystals of phosphorylated Ric-8A, which measured 50–250 µm along the unit-cell *a* axis and 5–20 µm in cross section (Fig. 1 ▸), were observed after 2–3 weeks of incubation time. Crystals of Ric-8A phosphorylated at Ser435 and Thr440 were larger in both length and cross section than those of unphosphorylated Ric-8A. Several cryoprotectants were tested. Crystals were harvested either with a PEG-based cryoprotectant (reservoir solution + 20% PEG 400) or an oil-based cryoprotectant (Paratone-N). We found that Ric-8A crystals were sensitive to glycerol and sugar-based cryoprotectants. The diffraction quality of the crystals deteriorated during storage in liquid nitrogen. Furthermore, all crystals dissolved in salt-based cryo-solutions. These observations were consistent with previous findings that penetrating cryoprotectants can increase the crystal mosaicity by displacing or replacing solvent in the crystal lattice (López-Jaramillo *et al.*, 2002 ▸). We found a solution of 20–25%(*v*/*v*) PEG 400 in reservoir solution to be a suitable cryoprotectant. PEG-cryoprotected crystals were generally isomorphous and diffracted to 2.2 Å resolution at conventional synchrotron sources using ∼12 keV energy. The crystals remained marginally iso­morphous after cryoprotection: the differences in unit-cell parameters are within 0.2–0.5% among these data sets, with mean values of *a* = 66.8 (0.3), *b* = 103.5 (0.2), *c* = 141.5 (0.6) Å (Table 1 ▸). We also used oil-based cryoprotectants, such as Paratone N, paraffin and Perfluoropolyether Cryo Oil (Hampton Research). In addition to their nonpenetrating properties, the oil cryoprotectants have the advantage that they reduce scattering and optical distortion during data collection (Riboldi-Tunnicliffe & Hilgenfeld, 1999 ▸). In our case, Paratone N provided excellent cryoprotection but resulted in shrinkage along all three unit-cell axes by 5–16% depending on the harvesting time (Zeng *et al.*, 2019 ▸). The anomalous data sets described here were collected from crystals of phosphorylated Ric-8A cryoprotected in PEG 400.

### 3.2. Anomalous signal analysis of Ric-8A crystals

In advance of collecting diffraction data, we used the *phenix.plan_sad_experiment* tool (Terwilliger *et al.*, 2016 ▸) to estimate the relationship between the *I*/σ〈*I*〉 of the data and the observed anomalous signal 〈*S*_ano,obs_〉. This indicator is proportional to the ‘useful’ correlation coefficient between observed anomalous differences and ideal anomalous differences (CC_ano_) generated by a Bayesian estimator on the basis of a diverse set of structures and data sets deposited in the PDB (Berman *et al.*, 2000 ▸),

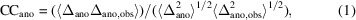

where Δ_ano_ is the ideal anomalous difference and Δ_ano,obs_ is the measured anomalous difference:

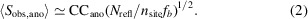




The tool was provided with the amino-acid sequence of residues 1–452 of Ric-8A, 4.2% of which are methionine and cysteine. Known or estimated parameters include the number of reflections, *N*_refl_, at the target resolution of 3.0 Å, the number of atoms that comprise the sulfur substructure, *n*_site_, and the second moment of the scattering factors of the anomalous substructure at the X-ray wavelength of 1.7712 Å, *f_b_*. For Ric-8A crystals, the maximum anomalous scattering from S atoms, *f*′′, is 0.8 e^−^.

At the target resolution of 3.0 Å, the anomalous signal 〈*S*_ano,obs_〉 is predicted to be below 8 assuming a maximum *I*/σ(*I*) of 100 and an estimated CC_ano_ of 0.56, corresponding to a 74% estimated probability of finding the anomalous sub­structure and an estimated figure of merit of phasing of 0.33. However, the probability and figure of merit are reduced to 26% and 0.27, respectively, at a target resolution of 5.0 Å. These estimates are made with the assumption that all sulfur atoms are highly ordered and fully occupied, and that the crystals do not suffer from radiation decay during data collection.

We concluded from the above analysis that assuming that the reflection data are collected accurately, as indicated by *I*/σ(*I*), and the atomic displacement factors of the S atoms are low, Ric-8A crystals would be expected to exhibit a measurable anomalous signal at a resolution limit of 3–3.5 Å (Tables 1 ▸, 2 ▸ and 3 ▸) depending on the choice of software used to scale and merge the 18 data sets.

### 3.3. Data collection and merging strategy

The aim of the S-SAD data-collection strategy is to measure a highly redundant intensity data set, affording full coverage of reciprocal space with accurate sulfur anomalous differences and minimal radiation damage. While the inverse φ data-collection mode minimizes the time interval, and thus differences in radiation-induced decay, between measurements of Friedel pairs, this strategy could not be implemented with the goniostat geometry and control software installed at the FMX beamline at the time that the data were collected. We therefore opted for very high redundancy afforded by measurement of rotation data from 14 single crystals (18 data sets) over oscillation ranges of 360–5760° in a helical data-collection mode to minimize radiation damage (Polsinelli *et al.*, 2017 ▸; Table 1 ▸).

The resolution of each of the data sets was determined according to the criterion implemented in *POINTLESS* and *AIMLESS* (Evans & Murshudov, 2013 ▸) whereby the resolution limit is defined as that at which CC_1/2_ falls below 0.3. By this measure, the diffraction limits of the 18 data sets ranged from 2.43 to 3.46 Å. This may have underestimated the resolution in data sets for which *I*/σ(*I*) > 2 in the highest resolution shell, where a steep fall-off in intensity and *R*_meas_ was observed in many of the crystals. Analysis of the anomalous differences in the individual data sets suggested that a more conservative limit of 3.4 Å would be appropriate. At this limit, the anomalous signal, |Δ_ano_ |/σΔ_ano_ (*d*′′/sig), did not exceed 1.0 for any of the data sets (Table 1 ▸). 14 of the 18 individual data sets exhibited poor CC_ano_ values that were not indicative of useful anomalous phasing power (Table 1 ▸). For these, the correlation of anomalous differences between half data sets, CC_ano 1/2_, was close to zero. Three data sets, 2_10, 2_14 and 2_15, were highly redundant and accounted for nearly half of the total observations in the 3.2–3.5 Å resolution range.

We used two strategies to generate merged and scaled Ric-8A data sets with the goal to extract sulfur anomalous differences of sufficient intensity and accuracy to reveal the sulfur substructure. The first of these, which employed the *CCP*4 program *BLEND* (Foadi *et al.*, 2013 ▸), was to identify clusters of data sets that would potentially yield the most accurate anomalous signals by optimizing isomorphism among the included data sets. Then, in *BLEND* synthesis mode, these data sets were scaled and merged in *AIMLESS* (Evans & Murshudov, 2013; Table 2 ▸). We also employed *phenix.scale_and_merge* (Terwilliger *et al.*, 2016 ▸) to process data-set clusters generated by *BLEND* without progressing though the synthesis stage. The second approach was to maximize redundancy by using all 18 data sets, employing *phenix.scale_and_merge* (Terwilliger *et al.*, 2016 ▸) to scale and weight individual data sets. At the same time, we were able to evaluate φ-weighted versus local scaling algorithms applied by *AIMLESS* and *phenix.scale_and_merge*, respectively. Both *BLEND* and *phenix.scaled-and-merged* exclude non-isomorphous or radiation-damaged images that would degrade anomalous signals.

**Figure 2:**
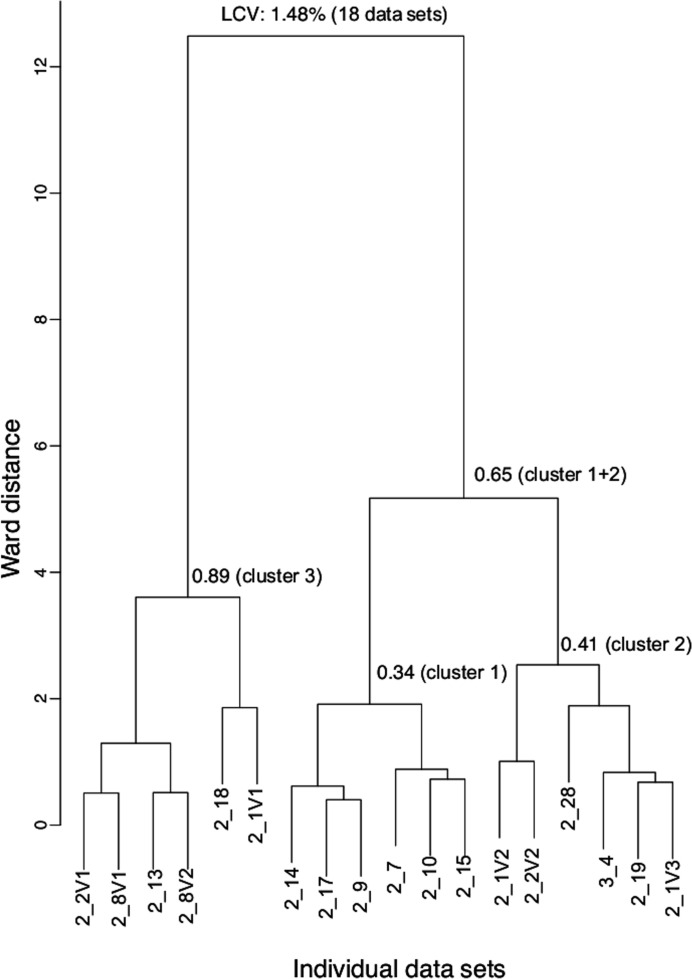
Data-set clusters generated using *BLEND*. The linear cell variation (LCV) is indicated for all 18 data sets and for each cluster.

Merging and processing of multiple data sets by *BLEND* was based on pairwise comparison of individual data sets to develop a hierarchy of data-set clusters, which is represented as a dendrogram (Fig. 2 ▸) based on the similarity of unit-cell parameters. *BLEND* analysis identified three subclusters, characterized by aggregate values of the linear cell variation (LCV) parameter in the range 0.34–0.89%. In contrast, the LCV for the entire data set was 1.48%, which corresponds to a maximum variation of 2.3 Å in the diagonal distances of the three unit-cell faces among the crystals in the data set. Execution of *BLEND* in synthesis mode evokes *AIMLESS* to scale and merge data within each cluster (Table 2 ▸). Monotonic changes in scaling *B* factors, typically over the range from −5 to −10, was consistent with the absence of significant radiation decay. Relative to the entire data set, and apart from cluster 2 and the 1+2 supercluster, clustering did not result in a significant reduction in *R*_meas_ or *R*_p.i.m._ despite the improvement in *I*/σ(*I*) for all but cluster 3, which includes many weak, high-resolution data. Importantly, none of the clusters appear to exhibit strong anomalous signals, as estimated by the slope of the normal probability plot of Δ*I*_ano_/σ(Δ*I*_ano_), where Δ*I*_anom_ = *I* ^+^ − *I* ^−^ (Evans, 2011 ▸). Likewise, no improvement is observed in the correlation of anomalous differences between half data sets (CC_ano 1/2_), which is not statistically significant for any of the data-set clusters. Using the criterion described above, *AIMLESS* set the high-resolution limit of the entire 18-crystal data set to 3.40 Å. The mid-slope of anomalous normal probability plot is 0.97, which is consistent with a marginal anomalous signal.

In contrast, when processed to a cutoff resolution of 3.24 Å with *phenix_scale_and_merge*, the data from clusters defined in *BLEND* and for the entire data set (Table 3 ▸) exhibited CC_ano 1/2_ values ranging from 0.29 to 0.74 and CC_ano_ values ranging from 0.49 to 0.72. The strongest anomalous signals were those from cluster 1, which includes three of the four data sets with the highest CC_ano_ values, and cluster 2, which includes the fourth (Table 1 ▸). Clusters composed of the largest number of data sets (1+2 and the set comprised of all data) exhibited the strongest anomalous correlation between half sets. We elected to retain a resolution limit of 3.4 Å for sulfur substructure calculations, in view of the observation that *d*′′/sig for cluster 3, at 1.04, is near the useful limit. At a *d*′′/sig of 1.45, the anomalous signal is much stronger for the full data set. However, due to the steep falloff in intensity with resolution, *d*′′/sig falls to 1.2 at 3.27 Å and to 0.8 at 3.0 Å.

### 3.4. Sulfur substructure determination

Calculations to extract the positions of native anomalous scatterers were conducted with data sets processed using *phenix_scale_and_merge*, as these exhibited the strongest anomalous intensity differences. We attempted substructure determination using the *phenix.hyss* submodule. which employs both dual-space completion and log-likelihood-based completion methods. *HySS* was executed without automatic termination in brute-force mode. The log-likelihood gain (LLG) scores for clusters 1, 2 and 3 and the 1+2 supercluster were 191, 115, 106 and 217, respectively, with corresponding correlation coefficients of 0.089, 0.084, 0.078 and 0.070. From five to seven anomalous scatterers were identified from each of these data sets, and in each case one or two of these corresponded to a sulfur-atom position in the refined atomic model of Ric-8A within an error threshold of 2.0 Å. Operating on the entire data set, *HySS* identified eight anomalous scatterers, of which five corresponded to correct sulfur sites, with an LLG score of 308 and a correlation coefficient of 0.13. We executed the *phenix.autosol* procedure on the entire data set, enforcing the inclusion of all data to 3.4 Å resolution during execution of the *HySS* tool. In this instance, *HySS* identified 45 anomalous scatterers with an LLG score of 1460 and correlation coefficient of 0.31. Of these positions, 40 corresponded to Ric-8A sulfur atoms.

**Figure 3:**
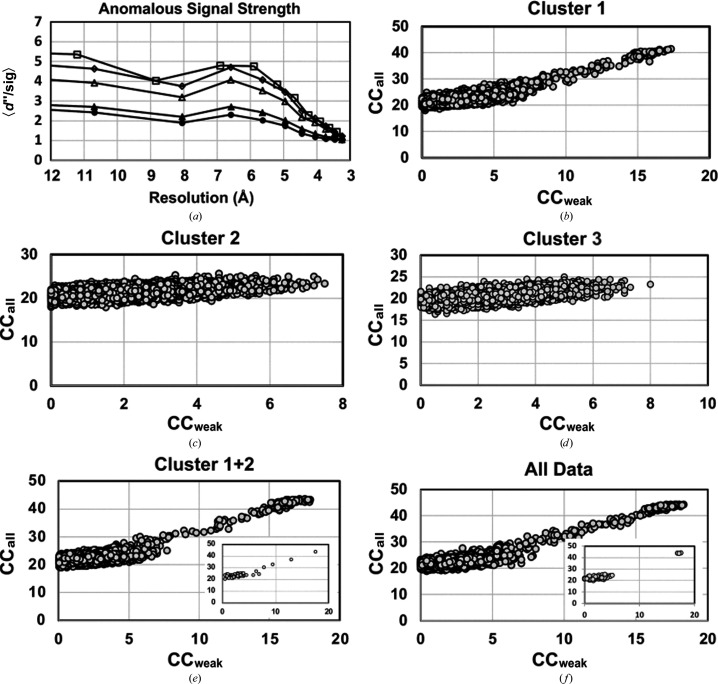
Anomalous substructure solutions from *SHELXC*/*D*. (*a*) Anomalous diffraction signal strength as a function of resolution, *d*′′/sig (Δ*F*/σΔ*F*), computed using *SHELXC*: empty squares, all data sets; empty diamonds, cluster 1+2; empty triangles, cluster 1; filled triangles, cluster 3; filled circles, cluster 2. (*b*)–(*f*) Correlation coefficients CC_all_ and CC_weak_ for 10 000 substructure solutions determined by *SHELXD*; the inset in (*f*) shows the distribution of solutions from 100 attempts.

We then turned to *SHELXC*/*D* (Pape & Schneider, 2004 ▸; Sheldrick, 2010 ▸) to determine the anomalously scattering substructure of Ric-8A. Based on *SHELXC* analysis, data within the 3.4–3.6 Å range, for which 〈|Δ_ano_|/σ(Δ_ano_)〉 (*d*′′/sig) ≃ 1.4, were set as the high-resolution shell for all clusters and for the full data set (Fig. 3 ▸ *a*). A substructure search using *SHELXD* was performed to test a maximum of 10 000 trials. For each solution, *SHELXD* computes CC_ano_ for all reflections (CC_all_) and for a set composed of the weak reflections (CC_weak_). In general, a bimodal distribution is expected for CC_all_/CC_weak_, in which correct, or nearly correct, substructure solutions form a cluster with relatively high values of C_all_/CC_weak_. Such a distribution was observed for the all-data cluster, cluster 1+2 and cluster 1, for which the highest-ranking solutions afforded CC_all_ = 44.1, CC_weak_ = 18.2, CC_all_ = 43.2, CC_weak_ = 17.1 and CC_all_ = 41.5, CC_weak_ = 17.3, respectively (Figs. 3 ▸ *b*, 3 ▸ *e* and 3 ▸ *f*). The top-ranked solutions for cluster 1, cluster 1+2 and the all-data cluster, respectively, included 36, 34 and 36 correct sulfur positions. Solutions for clusters 2 and 3 found only three and one, respectively, of the correct sulfur sites. Remarkably, *SHELXD* yielded several correct solutions for the all-data cluster and cluster 1+2 within 100 trials (Fig. 3 ▸ *f*). *SHELXC* operating on the all-data cluster merged and scaled using *AIMLESS* (Table 2) extracted anomalous differences with a *d*′′/sig of 0.6 at 3.4 Å, and subsequent execution of *SHELXD* yielded a monomodal distribution of CC_all_ versus CC_weak_ with maximum values of 25.0 and 9.6, respectively. Three of the 56 anomalous scattering sites corresponding to the latter highest-ranking solution corresponded to sulfur positions in the refined model. All of the clusters that afforded a correct solution included the large and highly redundant data sets 2_10, 2_14, 2_15 and 2_7, of which 2_7, 2_10 and 2_14 also exhibited relatively high CC_ano_ values (Table 1 ▸).

Operating on the all-data cluster processed with *phenix_scale_and_merge*, we employed the phase-retrieval program *PRASA* integrated in the *CRANK*2 suite to find the sulfur substructure of Ric-8A. *PRASA* conducted phasing trials at four high-resolution cutoffs ranging from 3.9 to 3.15 Å. The best solution emerged from refinement of solutions with a high-resolution cutoff of 3.4 Å, yielding a CC_ano 1/2_ (Karplus & Diederichs, 2012 ▸) of 25.9. Of the 40 S atoms in the asymmetric unit, *PRASA* correctly identified 35.

### 3.5. Structure determination

**Figure 4:**
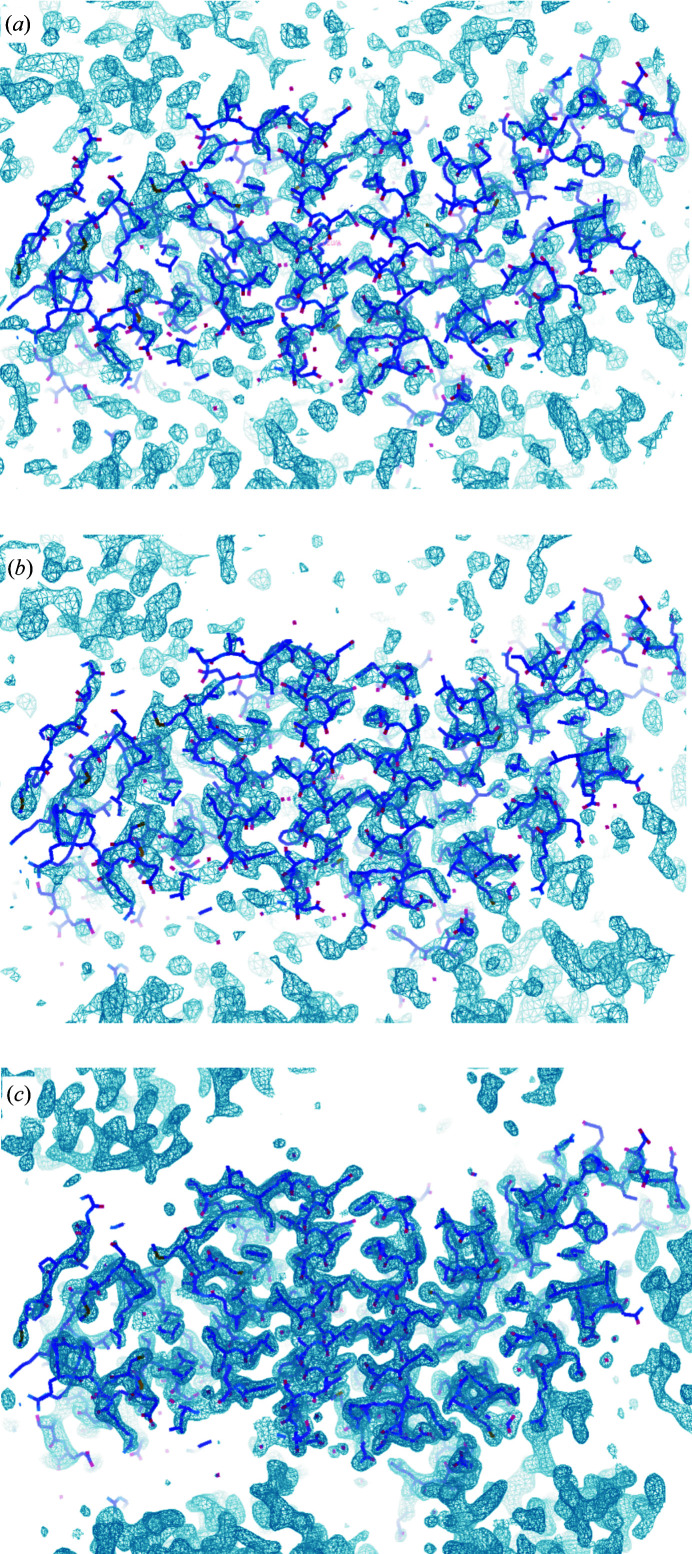
σ-Weighted 2*mF*_o_ − *DF*_c_ Ric-8A electron-density maps at successive stages in the phasing procedure. 3.4 Å resolution electron-density maps computed with SAD phases from the anomalous scattering substructure corresponding to the highest ranking solution determined by *SHELXD* using all 18 scaled and merged data sets are shown before (*a*) and after (*b*) density modification by solvent flattening. (*c*) σ-Weighted *mF*_o_ − *DF*_c_ electron-density map computed with native data measured at wavelengths of 0.979–2.2 Å with phases calculated from the final refined model. The refined Ric-8A model is shown in stick mode (C^α^ atoms in dark purple); electron-density maps were contoured at 1.5σ.

The structure of Ric-8A deposited as PDB entry 6nmg (Zeng *et al.*, 2019 ▸) was determined using the anomalous phases derived from the positions of the 36 S atoms identified by *SHELXC*/*D* as described above. The correct hand of the substructure was identified by density modification in *SHELXE*. We used the *ANOmalous DEnsity analysis* program (*ANODE*) in the *SHELXC*/*D*/*E* suite to compute the phased anomalous peak heights corresponding to S atoms in the 3.4 Å resolution anomalous difference map. These values ranged from 5.8σ to 16.9σ, with 29 sulfur atoms having values exceeding 8σ. Coordinates of the substructure atoms were submitted to the *phenix.autosol* pipeline for SAD phasing in *Phaser* (Adams *et al.*, 2010 ▸; Terwilliger *et al.*, 2009 ▸). Four additional sulfur atoms were located, yielding an overall figure of merit of 0.378 for the SAD phase set. Phasing and density-modification calculations yielded a promising solution with *R*-factor, map skew and model–map cross-correlation values of 0.2473, 0.10 and 0.79, respectively. Visual inspection of the electron-density map using *Coot* (Emsley *et al.*, 2010 ▸) shows continuous electron density corresponding to the predominant helical secondary structure of Ric-8A (Fig. 4 ▸ *a*).

**Figure 5:**
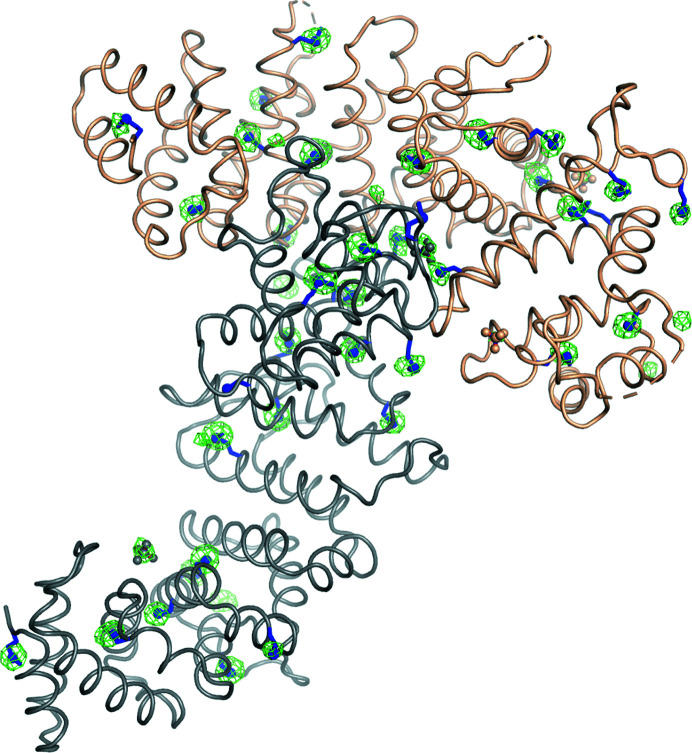
Anomalous difference electron-density map (Δ_ano_, α_calc_) computed with phases from the final coordinate set, showing the C^α^ trace for the two molecules in the asymmetric unit: molecule *A*, tan; molecule *B*, gray. Side chains of cysteine and methionine residues and sulfate ions are shown as stick models. The map is contoured at 4.5σ..

An initial model was constructed from the electron-density map computed with SAD phases from the sulfur substructure using the *AutoBuild* wizard (Terwilliger *et al.*, 2008 ▸). *AutoBuild* was able to trace helical fragments accounting for 16% of the asymmetric unit. After removing questionable residues, the main chains of both Ric-8A molecules in the asymmetric unit were retraced manually in a σ-weighted 2*mF*_o_ − *DF*_c_ map at 3.4 Å resolution using *Coot* (Emsley *et al.*, 2010 ▸), initially around the sulfur substructure sites and the *Autobuild* model. The initial phases were further refined and extended to 2.2 Å resolution using a native data set collected with X-rays of wavelength 0.979 Å (Fig. 4 ▸ *c*). Fragments of additional main chain were constructed after iterative manual model rebuilding and refinement with the *phenix.refine* tool (Afonine *et al.*, 2012 ▸). The registry of the sequence with respect to electron density was determined from the residues around the sulfur sites or bulky residues in both chains. NCS refinement was abandoned after the first few refinement cycles since the two molecules in the asymmetric unit (r.m.s.d. on C^α^ atoms of 0.718 Å between chains *A* and *B*) exhibited positional differences of >3 Å between corresponding C^α^ atoms and because several loop regions were disordered in chain *B*. An anomalous difference map computed with phases from the final model confirmed the 40 sulfur sites revealed in the anomalous sulfur substructure corresponding to nine methionine and nine cysteine residues from each of the two Ric-8A molecules in the asymmetric unit (Fig. 5 ▸). Four of the sulfur atoms in the sub­structure corresponded to sulfate ions derived from the crystallization buffer. Met426 was not located, possibly due to its flexibility in the structure. The final refinement statistics, indices of model quality and a description of the molecular architecture of Ric-8A and its relation to biological function are reported in Zeng *et al.* (2019 ▸).

## 4. Conclusions

With the advent of powerful beamlines and advanced phasing algorithms that combine sophisticated Patterson search procedures, direct methods and maximum-likelihood methods, experimental phasing using the anomalous intensity differences from native sulfur atoms has become routine. However, the method can present challenges for relatively small, moderately diffracting crystals that harbor large asymmetric units. Here, we have described our experience in the application of sulfur SAD phasing to determination of the structure of the G-protein-binding domain of Ric-8A, a 51 kDa protein that crystallized in an orthorhombic space group with two molecules in the asymmetric unit.

Crucial to the success of this project was the use of the NSLS II FMX (17-2) beamline as an X-ray source. Important attributes that contributed to accurate determination of anomalous intensity differences included an exceptionally high flux microfocus beam and a precision goniometer to position crystals within the 10 µm beam diameter, affording data collection in helical mode to minimize radiation decay. The helium-filled beam path and readout from the fast EIGER 16M pixel-array detector allowed the rapid recording of diffraction intensities with a minimum of air scatter.

We implemented alternative scaling/merging and substructure-search strategies encoded in publicly available program suites. In so doing, we approached the problem from the perspective of a routine user, making no attempt to modify existing software and, in most instances, did not explore program capabilities beyond those accessible though default options. Within this framework, we offer several observations that may be of use to other researchers who embark on S-SAD phasing of large asymmetric units of less-than-ideal crystals.

Firstly, high data redundancy is essential. This is a well recognized criterion for successful phase determination by S-SAD (Akey *et al.*, 2016 ▸). In the case of Ric-8A crystals, the multiplicity afforded by over eleven million intensity observations from 18 crystals, several of which were scanned over ten or more 2π rotations about the φ axis, proved to be critical. Importantly, the combination of multiplicity and unit-cell isomorphism proved to be decisive in defining the sulfur substructure. All of the data-set combinations that afforded the correct anomalously scattering substructure included the largest data sets from three highly isomorphous crystals that were aggregated in the same *BLEND* cluster. Secondly, we found that local scaling, as implemented in *phenix_scale_and_merge*, appeared to be more effective in extracting significant anomalous differences than the weighted φ-scaling implemented in *AIMLESS*. Finally, in our hands, it was possible to retrieve the anomalous scattering substructure using *SHELXD*, but not with the *phenix.hyss* tool.

To extract the anomalous substructure from crystals of Ric-8A, we collected X-ray data at 7 keV (1.7712 Å), at which the sulfur anomalous signal is significant while absorbance is manageable. However, modeling and experimental studies indicate that with a V-shaped detector geometry data collection at longer wavelengths approaching 3 Å, where *f*′′ is stronger, is advantageous if the cross section of the crystal and the surrounding cryoprotectant is low, in the neighborhood of 100 mm or less, where the effects of photon absorption are relatively low (Wagner *et al.*, 2016 ▸; Basu *et al.*, 2019 ▸). Beamlines BL-1A at the Photon Factory and I23 at Diamond Light Source are able to achieve such wavelengths, and several successful structure determinations of challenging targets using these facilities have recently been reported (Bent *et al.*, 2016 ▸; Parker & Newstead, 2017 ▸; Basu *et al.*, 2019 ▸). The FMX beamline can access wavelengths to 5 keV (2.48 Å), and we speculate that data collected at this energy might have reduced the requirement for high data multiplicity SAD phasing of Ric-8A crystals by virtue of the higher anomalous signal to noise that would be afforded at a wavelength closer to the sulfur *K* edge. Indeed, data sets collected with less than tenfold multiplicity on the I23 beamline at wavelengths ranging from 3.09 to 4.96 Å have led to successful structure determinations by native SAD phasing (Aurelius *et al.*, 2017 ▸; Langan *et al.*, 2018 ▸; Bent *et al.*, 2016 ▸).

## Figures and Tables

**Table 1:** Scaling parameters for single Ric-8A data sets Values in parentheses are for the highest resolution shell.

			Unit-cell parameters†										
Data set	Total range (°)	Resolution† (Å)	*a* (Å)	*b* (Å)	*c* (Å)	Unique reflections†	Multiplicity†	Completeness† (%)	*R*_meas_ † ‡ (%)	〈*I*/σ(*I*)〉†	CC_ano_ at 3.4 Å§	CC_ano 1/2_ at 3.4 Å§	|Δ_ano_|/σΔ_ano_ at 3.4 Å¶
2_1v1	360	30.0–3.27 (3.36–3.27)	67.04	103.96	142.08	15336	12.6 (9.2)	97.1 (71.1)	15.5 (92.6)	12.2 (1.7)	0.19	−0.09	0.7
2_1v2	360	30.0–3.24 (3.33–3.24)	66.49	103.64	141.01	15730	13.0 (11.5)	98.0 (82.5)	13.0 (91.3)	14.6 (2.6)	0.20	−0.04	0.7
2_1v3	360	30.0–3.39 (3.48–3.39)	66.70	103.47	140.90	13950	12.8 (10.9)	98.7 (91.0)	13.9 (91.4)	13.0 (2.1)	0.20	−0.06	0.7
2_2v1	360	30.0–3.32 (3.40–3.32)	66.82	103.66	141.44	14531	12.0 (10.5)	96.2 (52.6)	18.6 (100.2)	10.1 (1.5)	0.20	−0.07	0.8
2_2v2	360	30.0–3.41 (3.50-3.41)	66.77	103.71	140.90	13402	12.6 (8.6)	96.4 (55.3)	15.9 (95.5)	12.0 (1.6)	0.20	−0.08	0.7
2_7	1440	30.0–2.86 (2.94–2.86)	66.58	103.31	140.99	22912	50.6 (45.7)	99.1 (88.4)	7.4 (83.5)	50.0 (5.8)	0.45	0.21	0.8
2_8v1	1080	30.0–2.70 (2.78–2.70)	66.93	103.68	141.63	27004	13.3 (13.1)	97.7 (93.5)	6.5 (68.5)	27.1 (3.1)	0.26	0.01	0.8
2_8v2	1080	30.0–2.81 (2.88–2.81)	67.07	103.66	141.88	24457	29.7 (20.1)	95.7 (75.1)	11.2 (78.5)	29.4 (4.7)	0.23	−0.01	0.8
2_9	1080	30.0–2.64 (2.84–2.77)	66.33	103.43	140.71	24489	34.6 (21.2)	95.4 (89.1)	9.2 (82.8)	38.2 (3.5)	0.30	0.05	0.9
2_10	2880	30.0–2.97 (3.05–2.97)	66.39	103.36	141.23	21312	80.2 (75.1)	94.2 (73.7)	13.8 (79.2)	40.3 (0.9)	0.42	0.18	0.8
2_13	1440	30.0–3.25 (3.33–3.25)	66.93	103.61	141.96	15982	46.7 (37.1)	98.7 (84.0)	17.5 (93.0)	21.0 (3.6)	0.20	−0.05	0.8
2_14	5760	30.0–2.97 (3.05–2.97)	66.37	103.45	140.59	22225	134.8 (83.1)	99.7 (90.6)	15.9 (69.7)	42.0 (2.9)	0.45	0.18	1.0
2_15	3600	30.0–3.37 (3.44–3.37)	66.32	103.25	141.03	14122	100.1 (80.2)	95.6 (75.5)	13.1 (70.5)	34.3 (1.2)	0.21	−0.05	0.8
2_17	360	30.0–2.43 (2.50–2.43)	66.21	103.40	140.73	37043	12.8 (11.4)	99.6 (94.9)	7.3 (76.4)	23.8 (2.7)	0.25	0.00	1.1
2_18	1080	30.0–3.46 (3.55–3.46)	67.33	103.76	142.75	13414	37.1 (26.4)	97.6 (69.7)	16.2 (92.4)	22.0 (3.5)	0.20	−0.03	0.8
2_19	720	30.0–2.77 (2.84–2.77)	66.67	103.51	141.24	25093	26.6 (24.2)	97.7 (90.5)	11.0 (79.9)	24.4 (3.8)	0.30	0.04	1.0
2_28	1080	30.0–3.11 (3.19–3.11)	67.15	103.44	141.40	17574	37.8 (28.9)	96.2 (67.7)	13.1 (89.4)	29.9 (3.1)	0.20	−0.07	0.8
3_4	1080	30.0–2.81 (2.88–2.81)	66.75	103.40	141.33	24582	38.6 (35.5)	99.5 (94.5)	7.8 (79.5)	44.9 (5.2)	0.42	0.17	0.9

† Statistics were generated using *AIMLESS* from the *CCP*4 suite.

‡ *R*_meas_ = 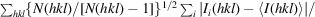 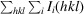 , where *I* *_i_* (*hkl*) is the *i*th observation of the intensity of reflection *hkl* and 〈*I*(*hkl*)〉 is the mean over *n* observations.

§ Statistics were generated using *phenix.anomalous_signal*. CC_ano_ = 〈Δ_ano_Δ_ano,obs_〉/(〈Δ^2^_ano_〉^1/2^〈Δ^2^_ano,obs_〉^1/2^), where Δ_ano_ is the ideal anomalous and Δ_ano,obs_ is the measured anomalous difference (*F* ^+^ − *F* ^−^). CC_ano 1/2_ is the anomalous correlation coefficient between half data sets.

¶ |Δ_ano_|/σΔ_ano_ values were computed using *SHELXC* (*d*′′/sig).

**Table 2:** *AIMLESS* scaling statistics for Ric-8A data-set clusters generated by *BLEND* Values in parentheses are for the highest resolution shell.

*BLEND* cluster	1	2	3	1+2	All data sets
LCV (%), MD† (Å)	0.34, 0.53	0.41, 0.64	0.89, 1.40	0.65, 1.01	1.48, 2.30
Resolution (Å)	31.13–3.45 (3.77–3.45)	29.89–3.61 (3.96–3.61)	31.41–2.54 (2.65–2.54)	29.89–3.61 (3.96–3.61)	31.3–3.40 (3.67–3.40)
Mean unit-cell parameters (Å)	*a* = 66.4, *b* = 103.4, *c* = 141.0	*a* = 66.8, *b* = 103.5, *c* = 141.2	*a* = 67.1, *b* = 103.7, *c* = 142.2	*a* = 66.8, *b* = 103.5, *c* = 141.2	*a* = 66.8, *b* = 103.5, *c* = 141.6
Unique reflections	13379	11765	28446	11764	14087
Average multiplicity	526.5 (527.5)	144.4 (146.9)	49.9 (1.2)	667.2 (665.9)	787.9 (805.0)
Completeness (%)	99.8 (99.8)	99.7 (99.6)	84.2 (15.3)	99.7 (99.2)	99.8 (99.5)
*R*_meas_ ‡	0.417 (0.612)	0.270 (0.346)	0.483 (21.095)	0.372 (0.458)	0.348 (0.480
〈*I*/σ(*I*)〉	33.2 (25.8)	38.0 (30.3)	15.6 (0.2)	45.6 (36.5)	58.5 (43.1)
*R*_p.i.m._ §	0.018 (0.026)	0.031 (0.039)	0.061 (13.273)	0.020 (0.024)	0.017 (0.024)
CC_1/2_ ¶	0.998 (0.997)	0.998 (0.998)	0.839 (0.065)	0.997 (0.999)	0.996 (0.994)
Anomalous completeness (%)	99.9 (99.8)	99.8 (99.6)	82.3 (7.1)	99.7 (99.2)	99.8 (99.5)
Anomalous multiplicity	282.5 (275.2)	78.2 (77.7)	499 (1.2)	360.4 (350.3)	423.7 (422.0)
Mid-slope, ANP††	0.77	0.89	0.73	0.91	0.97
CC_ano 1/2_ [Table-fn tfn10]	−0.175 (0.006)	−0.140 (−0.143)	−0.128 (−0.147)	−0.060 (0.048)	−0.092 (−0.217)

† MD is the largest variation across the diagonal distances (*D* *_ab_*, *D* *_ac_*, *D* *_bc_*) of the three unit-cell faces among data sets in the cluster.

‡ *R*_meas_ = 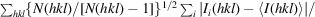 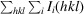 , where *I* *_i_* (*hkl*) is the *i*th observation of the intensity of reflection *hkl* and 〈*I*(*hkl*)〉 is the mean over *n* observations.

§ *R*_p.i.m._ = 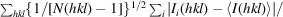 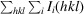

¶ CC_1/2_ is the correlation coefficient on corresponding intensities between half data sets.

†† Mid-slope of the anomalous normal probability plot of Δ*I*_ano_/σ(Δ*I*_ano_), where Δ*I*_anom_ = *I* ^+^ − *I* ^−^ (see Evans, 2011 ▸)

‡‡ CC_ano 1/2_ is the correlation coefficient between corresponding anomalous differences between half data sets.

**Table 3:** *Phenix.scale_and_merge* statistics for Ric-8A data-set clusters Values in parentheses are for the highest resolution shell.

Data-set cluster	1	2	3	1+2	All data sets
Resolution for scaling† (Å)	29.13–3.24	29.13–3.24	29.13–3.24	29.13–3.24	29.1–3.4
Total unique reflections/anomalous pairs†	29856/13790	29856/13790	29370/13786	29856/13790	25860/11894
Multiplicity†	240.3	69.6	56.9	294.3	380.5
Mean *I*/σ(*I*)†	126.2 (61.8)	74.58 (32.7)	60.6 (28.9)	147.6 (69.2)	173.9 (92.1)
CC_ano_ † ‡	0.70	0.51	0.49	0.72	0.72
CC_ano 1/2_ † §	0.60 (0.18)	0.29 (0.01)	0.303 (0.06)	0.669 (0.169)	0.742 (0.290)
*d*′′/sig at 3.4 Å¶	1.29	1.12	1.04	1.37	1.45
Unique anomalous pairs to 3.4 Å¶	11893	11894	11893	11844	11983

† Statistics were generated using *phenix.anomalous_signal*.

‡ CC_ano_ = 〈Δ_ano_Δ_ano,obs_〉/(〈Δ^2^_ano_〉^1/2^〈Δ^2^_ano,obs_〉^1/2^), where Δ_ano_ are the ideal and Δ_ano,obs_ are the measured anomalous differences (*F* ^+^ − *F* ^−^).

§ CC_ano 1/2_ is the anomalous correlation coefficient between half data sets.

¶ *d*′′/sig is the anomalous signal strength computed using *SHELXC*.
